# Molecular chemiluminescence imaging of misfolded proteins with a versatile and highly sensitive probe in neurodegenerative disease models

**DOI:** 10.1002/alz.090061

**Published:** 2025-01-09

**Authors:** Chongzhao Ran, Biyue Zhu

**Affiliations:** ^1^ Massachusetts General Hospital and Harvard Medical School, Boston, MA USA

## Abstract

**Background:**

Protein misfolding is a key pathological phenomenon driving neurodegenerative diseases that affect millions of people. Visualizing this misfolding process with smart imaging probes would greatly facilitate early diagnosis, etiology elucidation, disease progression monitoring, and drug discovery of neurodegeneration. Although numerous probes have been reported, several unmet needs still exist.

**Method:**

In this study, we demonstrated that both ***generic*** and ***precise*** visualization of misfoldons could be achieved with a highly sensitive chemiluminescence probe, ADLumin‐1.

**Result:**

For the ***generic*** aspect, we demonstrated that ADLumin‐1 was highly responsive when interacting with various misfoldons, including beta‐amyloid peptides, tau proteins, alpha‐synucleins, and TDP‐43, showing up to 128‐fold higher signal to noise ratio compared to gold standard dye Thioflavin T. Next, we explore the binding and the activating mechanism by molecular docking and proved ADLumin‐1 could non‐invasively visualize cerebral biomarker in transgenic mice models of Parkinson’s disease (PD), Alzheimer’s disease(AD) and amyotrophic lateral sclerosis (ALS). For the ***precise*** aspect, we explore the ***precise*** diagnostic value of ADLumin‐1 by using α‐synuclein, a key biomarker in PD. By combining with protein misfolding cyclic amplification (PMCA) technology, ADLumin‐1 enables selective detection of α‐synuclein in CSF at the femtomolar level. Finally, we showcased that ADLumin‐1 enables in situ monitoring of α‐synuclein misfoldons in transgenic mice by employing the bio‐orthogonal ChRET concept.

**Conclusion:**

Taking the advantages of dual facets of generality and precision, our findings could be broadly applied in the preclinical and clinical studies of neurodegenerative diseases.

